# Undergraduate medical education in Sierra Leone: a qualitative study of the student experience

**DOI:** 10.1186/s12909-018-1397-6

**Published:** 2018-12-07

**Authors:** Aniek Woodward, Danny McLernon-Billows

**Affiliations:** 1 Independent Researcher, Rotterdam, The Netherlands; 2 Independent Researcher, Liverpool, UK

**Keywords:** Undergraduate medical education, Medical graduates, Teaching staff, Experiences, Sierra Leone, Conflict-affected

## Abstract

**Background:**

Sierra Leone, a low-income and post-conflict country, has an extreme shortage of qualified medical doctors. Given the complex challenges facing medical education in this country and the need for context-specific knowledge, the aim of this paper is to explore the undergraduate medical education experience in Sierra Leone through qualitative interviews with recent graduates.

**Methods:**

In-depth interviews were conducted with purposively sampled junior doctors (*n* = 15) who had graduated from the only medical school in Sierra Leone. Additionally, semi-structured interviews were held with senior teaching staff at the School (*n* = 7). Interviews were conducted in October 2013. Results were thematically analysed.

**Results:**

The analytical framework consisted of four themes. *Medical school experiences* (Theme 1) were described as ‘stressful and tedious’ but also ‘interesting and enjoyable’. Various constraints were experienced linked to the *Medical school capacity* (Theme 2), including human (limited number of teachers, teaching skills), organisational (departmental differences, curriculum related challenges), physical (lacking teaching facilities on campus, transportation problems) and financial capacity (inadequate remunerations for teachers, most students receive scholarships). *Medical school culture* (Theme 3) was by some participants perceived as fearful and unfair. Findings suggest various *coping strategies* (Theme 4) were used at school (‘creatively’ hire extra teaching staff, teaching schedule upon availability of staff), staff (juggle multiple roles, teach flexibly), and student levels (comply with ‘hidden’ rules, negotiate teaching support from less qualified health personnel).

**Conclusions:**

This study has provided an insight into the student perspective on medical education in Sierra Leone. Numerous capacity related concerns were identified; which are unsurprising for an educational institution in a low-income and conflict affected country. While the School, staff and students have found creative ways to deal with these constraints, participants’ accounts of stress imply more is needed. For example, findings suggest that: students could be better supported in their self-directed learning, more effort is required to ensure basic needs of students are met (like shelter and food), and the power imbalance between staff and students could be addressed. Also better alignment amongst learning objectives and assessment methods will likely diminish student distress and may, consequently, reduce exam failure and possibly drop-out.

**Electronic supplementary material:**

The online version of this article (10.1186/s12909-018-1397-6) contains supplementary material, which is available to authorized users.

## Introduction

The global shortage and inequitable distribution of health workers severely limits the capacity of countries to delivery basic health services. Low-income, post-conflict countries like Sierra Leone are amongst the worst affected, particularly with regards to higher skilled cadres like doctors. The civil war (1991–2002) [1] and Ebola crisis (2014–2016) exacerbated health workforce shortages both directly (eleven doctors succumbed to the virus [2]), and indirectly by accentuating rural-urban maldistribution of health workers [3], accelerating their attrition through emigration [4], and temporary closure of health facilities [5]. Sierra Leone has the fifth lowest doctor to population ratio in the world at just 2.4 per 100,000 people [6].

Numerous national policies in the post-conflict and post-Ebola periods have highlighted the importance of strengthening the health workforce in Sierra Leone through reforms to pre-service training [7–10]. Sierra Leone’s health training institutions are currently challenged by: underfunding, shortages of qualified educators, inadequate infrastructure and teaching materials [11]. Sierra Leone’s only medical school has a 45% dropout rate over the six-year degree [12], four times higher than the international average [13]. These issues affect Sierra Leone’s ability to produce enough adequately trained doctors and ultimately undermine health system stability.

Policymakers face a challenge developing evidence-based interventions to address the issues facing medical training in Sierra Leone as there is a lack of literature relevant to their context or need. The vast majority of medical education research is produced in high income settings, and of the available sub-Saharan African (SSA) literature, two thirds comes from just three countries (Uganda, Nigeria and South Africa) [14]. Chen et al. published a comprehensive overview of the challenges faced by medical schools in SSA, and steps being taken to expand capacity [15]. They did not seek the opinions of students or graduates however, who are likely to have different perspectives to the educators and faculty consulted by the study.

It is accepted that medical curricula should be responsive to the needs of students, institutions, and society [16]. Medical education research therefore should also reflect these three perspectives. Understanding the student perspective is a key aspect of quality improvement in higher education [17].

To our knowledge, only three studies have attempted to comprehensively explore the student experience of undergraduate medical education in SSA countries: South Africa [21], Ethiopia [22] and Kenya [23]. These studies found that interviews and group discussions with students and recent graduates provided valuable insights about how to strengthen curricula. Mengistu (2017) for example offers cautionary lessons for Sierra Leone about the impact that rapidly expanding student intake has on the quality of training [22]. In general the findings of these studies are highly context-specific and highlight the importance of creating local solutions to local problems. Given the complex challenges facing medical education in Sierra Leone and the need for context-specific knowledge, the aim of this paper is to explore the undergraduate medical education experience in Sierra Leone through qualitative interviews with recent graduates.

### Background on medical education in Sierra Leone

The College of Medicine and Allied Health Sciences (COMAHS) was established in 1988 and since 2005 is part of the University of Sierra Leone. COMAHS is the only Medical school in Sierra Leone that educates doctors. Its medical undergraduate curriculum spans six years and is based on the West Africa Health Organisation (WAHO) harmonised curriculum [18]. Table 1 provides an overview of the latest Bachelor of Medicine and Bachelor of Surgery (MBChB) curriculum.

**Table 1:** Overview of MBChB curriculum at COMAHS (Author compilation, based on information from curriculum in 2014 [18])

Year 1	Year 2	Year 3	Year 4	Year 5	Year 6
Basic medical sciences (anatomy, embryology-histology, physiology, biochemistry & genetics cell-biology, community medicine, integrated clinical skills, psychology, medical sociology, French)		Para-clinical sciences (laboratory medicine, integrated clinical skills, pharmacology, community medicine, French)	Clinical sciences (medicine, surgery, paediatrics, obstetrics & gynaecology, community medicine, professionalism and ethics, French, research project, electives)		

Basic sciences teaching mostly takes place at the campus in Kossoh Town (a coastal suburb about ten miles east of Freetown) and Connaught Hospital (tertiary hospital in central Freetown). Clinical rotations are predominantly in three public teaching hospitals in Freetown (Connaught Hospital for medicine and surgery, Princess Christian Maternity Hospital for obstetrics & gynaecology, and Ola During Children’s Hospital for paediatrics). Students can also gain practical experience in private hospitals and clinics in the capital. Most medical students precede their undergraduate course with one or two years of pre-medical education [19]. All graduates are required to undergo a two-year internship programme before gaining full registration with the Sierra Leone Medical and Dental Council and being able to apply for further education in a specialised area of medicine.

## Methods

### Recruitment of participants

Junior doctors were purposively selected based on maximum variation (mixture of men/women, years of graduation and career stages (house officer, medical officer, resident). This type of sampling, which originated from grounded theory, identifies “information-rich” cases [20]. Doctors who graduated from the College of Medicine and Allied Health Sciences (COMAHS) from 2002 onwards (i.e. post-civil war) were eligible for participation in the study.

Personal contacts of an international non-governmental organisation were accessed first to identify potential junior doctors for participation, followed by further snowballing sampling [21]. This process of recruitment generated a cohort of 48 junior doctors. Those with characteristics needed to achieve maximum variation were invited by the lead researcher (AW) to participate by phone or email. The aim was to achieve a sample size of about 10–15. In total 28 doctors were approached and 15 agreed to participate. Not answering the phone or non-response via email after initial approach were the main reasons for not being interviewed.

Secondly, key informants were purposively sampled to get a range of perspectives from senior teaching staff at COMAHS (i.e. mixture of male/female staff from various medical departments). A similar recruitment strategy as with junior doctors was applied.

### Methods and process of data gathering

*In-depth individual interviews* were conducted with junior doctors (*n* = 15), using a life-history approach. This approach is understood as personal narratives situated in time and context, and was found applicable in a recent publication about career experiences of health workers in post-conflict settings [22, 23]. Participants were asked to complete a lifeline chart at the start of the interview to aid reflection on past experiences. This chart recorded key family events (births, deaths, illnesses), places lived, educational and employment history. Completed charts and a topic guide helped structure individual interviews. This topic guide was based on the literature [4, 24, 25] and experiential knowledge. Topics from the guide focused on in this paper concern experiences (e.g. ‘What did you enjoy most and what did you enjoy least in your experience as a medical student?’) and financing of medical education (e.g. ‘How did you fund your medical education?’). A more detailed interview guide is found in Additional file 1. A pilot focus group was conducted with three medical students from Sierra Leone to test the life-line chart and trial relevant parts of the interview guide for junior doctors. No changes to the content of the interview was necessary as students understood all questions. Additionally, follow up interviews and diaries were used to explore evolving career experiences of junior doctors. These results were not the focus of this paper and are described in a related publication [26].

*Semi-structured individual interviews* were held with senior teaching staff (*n* = 7). The topic guide was based on the literature [27–31] and experiential knowledge. Topics covered in this paper include their roles in medical education, and perceptions on financing (e.g. ‘How is medical education funded in Sierra Leone?) and quality of medical education (e.g. ‘How would you describe the quality of medical training provided at COMAHS?’). A more detailed interview guide is found in Additional file 1.

The place and time of every audio-recorded interview was negotiated with each participant and the researcher. All participants provided written informed consent prior to the interviews. Most interviews were conducted face-to-face during a fieldtrip to Freetown in October 2013.

### Data analysis

All interviews were transcribed and analysed with NVivo 10.2.2. © QSR International (a qualitative computer software programme) by the lead researcher (AW). The lead researcher is not a medical doctor but a specialist in health systems research with an interest in health worker experiences. Thematic analysis was applied to explore relationships and ‘themes’ [32] across the interviews. After familiarisation with the transcripts, data was initially coded and further refined, ordered, reordered, categorised and themed until a final coding framework was identified. A code had to mentioned by at least two participants to be considered a sub-theme. This matrix was then applied to all transcripts.

Various recommended approaches [33] were considered to increase validation of the interpreted data. Firstly, through constant comparison differences and similarities were explored. Differences by gender and cohort year were the main foci. Efforts were made to search for ‘deviant cases’, which are those that might disconfirm emerging themes [34]. Secondly, single counting was applied to indicate how strong the evidence of experiences was amongst the study cohort [34]. Lastly, initial findings were presented to project supervisors to check if results were aligned with the study objectives.

In the following section many direct accounts are included, which is particularly important in the life-history approach [35]. The source of each account cited is indicated as junior doctor (JD) or senior teaching staff (TS), accompanied by a randomly assigned number for each, to illustrate the range of responses. Background information such as gender or cohort of JD is provided where relevant.

## Results

### Participants

Fifteen junior doctors who graduated between 2006 to 2015, gave consent for the interview study together with seven senior teaching staff at the medical school in 2013 (see Table 2). These dual perspectives generated an in-depth understanding about undergraduate medical education in Sierra Leone.

**Table 2:** Study participant socio-demographic information

Sample	Characteristic	
*Junior doctors (n = 15)*	Age	29 years (average); 24–35 years (range)
	Gender	60% male; 40% female
	Graduation year	2015 (n = 1), 2013 (n = 2), 2012 (*n* = 4), 2011 (*n* = 2), 2010 (n = 1), 2009 (*n* = 3), 2008 (n = 1), 2006 (n = 1)
	Marital status	single/dating/divorced (60%); married (40%)
	Religion	53% Christian; 47% Muslim
*Senior teaching staff* ^*b*^ *(n = 7)*	Gender	72% male; 28% female

^a^Includes those formally employed by COMAHS and those teaching on a voluntary basis

### Themes

The analytical Framework consisted of four key themes: 1) Medical school experiences; 2) Medical school capacity; 3) Medical school culture; and 4) Coping strategies. Table 3 provides an overview of these themes and their sub-themes.

**Table 3:** Overview of themes and sub-themes

Theme 1. Medical school experiences	
1.1 Stressful and tedious	
1.2 Interesting and enjoyable	
Theme 2. Medical school capacity	
2.1 Human capacity	
2.1.1 Number of teaching staff	
2.1.2 Time dedicated to teaching	
2.1.3 Teaching skills of staff	
2.2 Organisational capacity	
2.2.1 Departmental differences in organisational capacity	
2.2.2 Curriculum related challenges	
2.3 Physical capacity	
2.3.1 Impact of civil war on the campus and teaching hospitals	
2.3.2 Travel to and from the campus	
2.3.3 Facilities at the campus and teaching hospitals	
2.3.4 Learning resources	
2.3.5 Teaching aids and medical equipment	
2.4 Financial capacity	
2.4.1 Remunerations for teaching staff	
2.4.2 Scholarships for students	
Theme 3. Medical school culture	
3.1 Culture of fear	
3.1.1 Fear of teaching staff during bedside and classroom teaching	
3.1.2 Fear of failure during exams and assessments	
3.2 Culture of perceived unfairness	
3.2.1 Rumours of impartiality in scholarships	
3.2.2 Perceptions of favouritism in class and examination	
Theme 4. Coping strategies	
4.1 School level	
4.1.1 ‘Creatively’ hire extra teaching staff	
4.1.2 Base teaching schedule upon availability of teaching staff	
4.2 Staff level	
4.2.1 Juggle of multiple roles	
4.2.2 Teach flexibly	
4.3 Student level	
4.3.1 Comply with ‘hidden’ rules	
4.3.2 Negotiate teaching support from less qualified health personnel	
4.3.3 Get by with self- directive learning	
4.3.4 Cooperate with other students	
4.3.5 Student union acting as advocate for students	
4.3.6 Rely on financial and moral support from family and friends	

#### Theme 1. Medical school experiences

The experiences by junior doctors during their time in medical school was described in two ways; e.g. on the one hand as stressful and tedious, and on the other interesting and enjoyable.

##### Stressful and tedious

Stress’ or ‘difficult’ is often the initial response of junior doctors (13/15) when asked “What is your experience of medical education?” Some specific descriptions expressed were: “COMAHS was stress” (JD14), “it [medical school] was hectic” (JD13), “there were lots and lots of challenges [in the medical school].” (JD7), “Medical school of itself is very stressful” (JD11), “I’m not sure if there’s a University in the world that is more difficult than COMAHS” (JD15). These explicit reports of stress cut across all undergraduate years. A reason for this negative experience was the perceived high workload for students (5/15):

“I’m frustrated by the overwhelming work we have to do in our current year, which is the final year of our course. We are to write a dissertation, we are to prepare for our final year exams…I am also doing some part-time work.” (JD1)

Half (7/15) of the participants depicted medical school, particularly the basic medical sciences (the first three years), as ‘tedious’ or ‘boring’: “The first years of medical school were kind of boring” (JD4).

##### Interesting and enjoyable

Medical education, however, was also described as enjoyable and interesting. For five interviewees this happened at the start of clinical teaching in Year 4. Direct patient contact was considered an important motivator (6/15). Contact with patients allowed students to put theory into practice (2/15):

“And I really enjoyed those ones, the clinical years. Because I have ‘my hands on the job’. I can see patients, I can see people, relate to what I know.” (JD13)

In addition, patient contact stimulated identity formation (5/15):

“But when I was in [the] clinical [years] you have to go to the wards and see patients. You start feeling like a doctor you know.” (JD7)

Junior doctors (4/15) found exposure to patients in abundance during their undergraduate medical education:

“In Sierra Leone there is an abundance of patients with a wide range of symptoms, so you get to see a lot more of the illnesses here and I have access to them. Because people are always willing to let you examine them and learn from them.” (JD4)

Besides patient contact, another reason why medical school seemed to be a positive experience, was the respect received by the trainees (2/15) about their future profession in the community:

“It [medical school] is good because when you’re living in a community and someone says ‘oh she’s doing the doctor course’, you’re respected from that stage.” (JD12)

While the time in medical school became more progressively motivating, accounts of stress and challenges remained. Two themes seem particularly related to stress: several limitations in institutional capacity and culture.

#### Theme 2. Medical school capacity

Various constraints were experienced by junior doctors and lecturers during undergraduate medical education, which can be categorised as human, organisational, physical and financial capacities.

##### Human capacity

This section reports on a number of important issues in relation to teaching staff.

*Number of teaching staff*

Lecturers (7/7), “There are only three of us in the XX department” (TS14), and junior doctors (5/15), “…they only had one teacher in the whole XX department.” (JD10), alike reported staff shortages.

One reason seemed to be closely linked to the civil war that took place 1991–2002, which resulted in a lack of teaching staff according to lecturers (4/7): “Most of the lecturers left during the war actually.” (TS16) Teaching staff (3/7) expressed that some faculties in the School were disproportionally affected:

“COMAHS then [before the war] had a very good staff profile…And we lost that because of the war. Particularly for the XX faculty, which had a lot of support from WHO. The WHO was bringing in specialists from the sub-region and we lost that kind of support because people were not willing to come [to Sierra Leone]. It [is] still affecting the College.” (TS12)

There has been a gradual rise in medical school entrants after the civil war in order to increase the number of doctors in Sierra Leone. Although the quantity of teaching staff has grown since as well, this was regarded insufficient by teaching staff (7/7): “We have more staff now [in 2013] although [it is] still not sufficient.” (TS20)

Eleven Sierra Leonean doctors lost their lives during the Ebola outbreak (2014–2015). Follow-up interviews with junior doctors (4 /11) illustrated that the crisis included staff from COMAHS:

“Now there are so many who have passed away [because of the Ebola outbreak]…I knew all of them. Most were my lecturers in fact.” (JD15)

In addition, the shortage of teaching staff affected the time the doctors were able to commit themselves to high quality teaching.

*Time dedicated to teaching*

Both students (7/15) and staff (6/7) found lecturers had a lack of time to teach:

“The teaching was really not that effective…Very few lecturers…Most of them were much involved in their private work. So they didn’t have much time you know to lecture.” (JD8)

“The lecturers are not many and they have other commitments.” (TS20)

A couple of trainee doctors (2/15) reported that teaching staff is overworked:

“We don’t have many lecturers. And even the ones that we have, they have a lot on their plates. They come when they are free.” (JD9)

This last comment indicates that teaching staff did not always attend scheduled classes. There were various accounts (5 /15) by the junior doctors of lecturers being late or absent: “They [the lecturers] are usually late. And occasionally they are absent.” (JD11) Another added that some lecturers lacked the knowledge of how best to teach:

“And at times you might find that maybe out of two lecturers, one lecturer is not coming. And the other lecturer is very boring. He doesn’t know how to teach well.” (JD1)

This account also indicated that some staff were perceived to have limited teaching skills.

*Teaching skills of staff*

Junior doctors (7/15) mentioned a wide variety of teaching skills by lecturers at COMAHS: “…some people are born lecturers and there are people that just try what they can do” (JD3). Various reasons were provided by the young doctors (7/15) why the teaching skills by some lecturers were regarded less effective than others. A comment by one doctor sums up three reasons:

“Because either the lecturers were too busy. They asked you to read from the books or we [students] had a few lecturers who [we] were afraid of being victimised.” (JD10)

Some doctors (3/15) commented on the traditional didactic teaching style of certain teaching staff:

“And one or two lecturers also like to dictate notes. That I think is outdated for our standard. They explain a bit and then give us some notes.” (JD1)

Deficiencies in teaching skills by selected lecturers made asking questions in class difficult, according to several trainee doctors (3/15):

“You can’t really ask a lot of questions in class because you just want the class to be over without getting into trouble and get out of there. So we had a few of those [lecturers].” (JD13)

##### Organisational capacity

The medical school seemed to have curriculum-related challenges and differences across the four main departments (e.g. Medicine, Surgery, Paediatrics, and Obstetrics & Gynaecology).

*Departmental differences in organisational capacity*

Attendance varied between departments. A few trainee doctors (2/15) highlighted the importance of attendance monitoring for learning. The administration of departments with the severest staff shortages appeared especially affected, according to junior some doctors (5/15):

“I mean the postings are well organised [Department X] because there are a lot more lecturers than in the [Y] Department.” (JD11)

Overall, it looks like the medical school struggles with the delivery of its curriculum.

*Curriculum related challenges*

The teaching schedule in the School seemed to be ad-hoc. One doctor (JD8) talked about not having a syllabus until year four and another (JD6) questioned the existence of a solid curriculum, which was confirmed by a teacher (1/7):

“But honestly speaking I think…the [teaching] schedule [at COMAHS] is crazy and I think that it should be better organised….it should be more set and not depend on the whims of the lecturers.” (TS18)

A third of junior interviewees (5/15) commented that exams during basic science years took place annually instead of after each semester, which meant they were more demanding:

“We don’t have a semester system. We have an annual exam at the end of the year. And we have few tests during the year…But because of the annual system, some students relax the initial part of the year. At the end of the day, it becomes very stressful.” (JD1)

Three trainee doctors (3/15) explained working at a higher level than what is expected for their stage of their study:

“And what is expected of the students is also too much. The books we use, some of them are meant for post-graduate studies. They are reference books, yet our lecturers expect us to know them.” (JD2)

##### Physical capacity

There were perceived limitations in physical capacity at COMAHS, including its facilities, such as at the campus and in teaching hospitals, including the relevant learning and teaching resources.

*Impact of civil war on the campus and teaching hospitals*

One doctor who started premedical education during the civil war recalled not being able to use the COMAHS campus at Kossoh Town, which is a coastal village about ten miles east of Freetown: “Because for example our campus…was where the rebels were staying. So they [the rebels] destroyed a lot of it [the campus].” (JD4) Around 2001 for example, the campus started to function as a student dormitory, while teaching still took place elsewhere: “All the lectures were here in town. People would normally go there [to the campus] if you wanted to escape the noise [of family life] at home.” (JD14) Connaught, the main teaching hospital, was not utilised for some time because it needed to be reconstructed:

“…we were like the post-war people after the war…Even at that time Connaught was being rehabilitated [reconstructed]. So they [medical school] were not using Connaught… By the time we were in fourth year, they had reopened Connaught and they started using Connaught.” (JD3)

“Particularly at Connaught and the other tertiary hospitals…[medical] equipment deteriorated, got missing, destroyed, and replacing them [medical equipment] has been a huge task [after the war].” (TS12)

Once the campus and teaching hospitals were rebuilt, the interviewees reported the inconvenience of travel between the campus and hospitals.

*Travel to campus and teaching hospitals*

Almost half (6/14) of junior doctors interviewed requiring travel to the campus (one did not need to travel as this person started training during the war when the campus was not in use) reported difficulties in going from the Kossoh Town’ campus to the center of Freetown, where the teachings hospitals were based.

Although students were served by a school bus, the arrangements were found unreliable (4/15):

“With the traffic and everything you could spend more than 3 hours [from Kossoh Town to center of Freetown]…. Initially when I came to COMAHS we had buses. Then the buses went out of order. Now I think the administration have a bus but that bus is meant to move twice a day.” (JD9)

“…the bus is almost always late.” (JD12)

Few (2/15) trainee participants highlighted transportation problems also affected lecturers:

“I don’t think there are enough buses to take the lecturers to the place [campus]. Or even if there are, which I doubt, I don’t think it will be at a time convenient or matching with the lecturers’ time.” (JD6)

Besides transportation inconveniences, there were also challenges with regards to the campus’ facilities.

*Facilities at the campus*

One doctor (JD9) commented that the Kossoh Town’ campus was frequently closed to improve its facilities. Several (6/15) interviewees described that the campus had an unreliable supply of water and electricity: “At Kossoh Town, the building is there, but no electricity, no water.” (JD9) Others (2/15) also reported a lack of food: “There was no food on campus.” (JD14) Another (JD12) commented that food was available at the campus but too expensive. Two female doctors (2/6) feared for their personal safety on campus: “It wasn’t safe. It was just two buildings in a bush…Because when I was there it wasn’t fenced.” (JD13). The library at the Kossoh Town campus was either absent or regarded as dysfunctional according to participants (4/15). Positively, few (2/15) junior doctors mentioned how some of the apparent difficulties had been addressed over the years.

“…now they [medical students] have light and then I think the water system is improved now…It’s only now that the surrounding village [of the campus] has something like supermarket or minimarket where they [students] are able to buy things.” (JD9)

Several (3/15) also provided positive comments about campus life, including “fun” and a family feel (JD8) and as a place to “escape the noise at home” (JD14).

*Learning resources*

Half of the junior interviewees (7/15) mentioned that textbooks were financially and physically difficult to access: “The books, they are very expensive. And even if you have the money to buy them, you can’t get them so easily.” (JD12) In addition, they (3/15) experienced physical barriers to log onto computers:

“For example, for like many years we [medical students] paid for ICT, for technology, computer services [at COMAHS]. But there were no computer services available.” (JD5)

“It’s not easy for someone…to have a computer. I only had a computer towards my fifth year.” (JD12)

The Internet was regarded by interviewees (3/15) as “very slow” (JD1) and “expensive” (JD14). Positively, a 2015 graduate observed an increase in Internet use on mobile phones amongst medical students.

*Teaching aids and medical equipment*

A third of junior participants (5/15) reported a lack of teaching aids and medical equipment, e.g. anatomical models (JD7) and a diagnostic set, (JD12), as unobtainable in Sierra Leone. A teaching staff member (TS10) believed that having limited medical equipment during medical education means that students are better prepared to work outside of Freetown, where equipment is even scarcer.

Medical students who go for electives outside of Sierra Leone may see those medical instruments and procedures, which are unavailable in teaching hospitals in their home country. For example, two junior doctors (2/15) commented on seeing an endoscopy performed for the first time when abroad.

“We were able to see things that we do not have here [Sierra Leone]. I think over there [West Africa] was the first time I saw an endoscopy done. And after then, it’s only when I became a house officer, that I saw it [endoscopy] being done at Choitrams [a private hospital in Freetown].” (JD9)

Positively, accounts by a junior doctor (1/15), “So now we’ve got learning aids like projectors and slides, which we didn’t have when I was in Medical School” (JD4) and a lecturer (1/7), “Since 2005 when I do my lectures, I would prepare slide shows like power point presentations. So things have definitely changed” (TS14),

indicate there has been some progress in relation to teaching aids.

##### Financial capacity

COMAHS as an institution is funded by the Ministry of Education through the University of Sierra Leone and the Ministry of Health and Sanitation funds some students who attend COMAHS via scholarships. In this section results on remunerations for teaching staff and scholarships for students are reported.

*Remunerations for teaching staff*

Limitations of financial resources for the medical school meant several teachers (4/7) interviewed for this study received no compensation for their lessons: “They [COMAHS] don’t pay me. It’s my giving back [to COMAHS]” (TS18). One lecturer was offered a salary but “opted to work for free” (TS10). Those that were remunerated (3/7) agreed the salary was inadequate: “It [ the salary] might not be enough but they [the medical school] are paying me.” (TS16)

*Scholarships for students*

Almost all junior doctors (14/15) received a scholarship from the Sierra Leonean Government at some point during medical school, which covered most of the tuition fees. Several doctors (6/15) received this government funding throughout their entire medical education. Others paid the first year (6/15) or three years (2/15) themselves, often through family sponsorship, before being granted a scholarship. One doctor paid for his medical education through parental support as he was not eligible for a scholarship as a foreign student.

Three reasons (merit, household income, and nepotism) were provided by junior doctors (4/15) that helped them to receive these scholarships.

“Yes [I got a scholarship] from the start [of medical school] because when I did my school leaving exam I did very well and so I got an immediate scholarship from the government.” (JD13)

“Then if you’re coming from like a working-class family they [at the medical school] try to make sure that the scholarship goes to those who really can’t afford it.” (JD4)

Examples of nepotism can be found below under ‘3.2 Culture of perceived unfairness’. If a student had to repeat a school year, he or she was required to reapply for the scholarship, although one doctor (JD11) in this study repeated twice, but each time was given the scholarship again the following year.

One young doctor (JD5) believed that they could have afforded medical education without the scholarship, while others (2/15) were less positive:

“I don’t think so because my dad is sick so it all depends on my mum, so no. I don’t think I would have [been able to afford medical school] otherwise.” (JD10)

One junior doctor (JD8) commented that because the yearly tuition fee was perceived expensive, considering the socio-economic status of people in Sierra Leone, this might deter some from entering medical school.

Even when tuition fees were largely covered via the government scholarship, some (5/15) still found it a stretch to pay for books, food, and ‘additional charges’ to the medical institution (i.e. laboratory work, sports, computer services). One doctor summed up clearly the various financial challenges faced by medical students with less financial means:

“Some students might have a scholarship and 95% of fees are paid for. And that 5% still becomes a problem…Books, photocopy, you need to move from one place to the other, from one hospital to the other…and you need to buy food. And food is a bit expensive…So some students find this really difficult.” (JD5)

#### Theme 3. ‘Medical school culture’

Accounts by junior doctors suggest the culture at COMAHS can be described as fearful and unfair.

##### Culture of fear

Fear of teaching staff during bedside and classroom teaching and of failure during exams and assessments were frequent comments during interviews.

*Fear of teaching staff during bedside and classroom teaching*

Junior doctors (6/15) described feeling fearful during bedside and classroom teaching. Female graduates (4/6) particularly raised this concern, although male doctors were also affected (2/9).

“It was when you’re like doing your bedside teaching. In front of the nurses, and the patients…And the lecturer would be there asking questions you don’t even imagine you would find answers to. And sometimes *he* would go and get angry. *He* would start shouting. It would leave the patient very ashamed. But they do that…They force you to learn by embarrassing you.” (Female doctor)

“So now you go to the ward…They [lecturers] just ask you questions…Questions that you’re actually not supposed to know…So you’re actually not being taught. You’re in fact being threatened. You’re being intimidated.” (Male doctor)

The use of fear tactics seems particularly ingrained in the medical school culture in Sierra Leone as reported by a female doctor’s comment in which she compared her experiences in Sierra Leone with another West African country:

“And here [Sierra Leone] we’re always afraid of the consultants or professors, you’re always afraid, compared to there [in another West African country]. Well I guess it’s the way they interact with us. Because all of the time they [teaching staff] are shouting.” (JD9)

The same doctor explained that some clinicians behaved more positively towards her once she graduated from medical school.

*Fear of failure during exams and assessments*

This culture of fear was also experienced during written and clinical exams. When recalling their days as medical students, exam periods were perceived as particularly stressful due to a fear of failure (4/15):

“So they [lecturers] almost always instil fear in you. So the atmosphere in which you study is almost always ‘oh am I going to make it this year?’ Even if you study and study and study, you are always afraid until the exam results are out.” (JD12)

Junior doctors (6/15), “And it [medical school] was very challenging because the failure rate [for exams] was quite high” (JD10), as well as a lecturer (1/7), “It’s only maybe about less than 30% who go through COMAHS without repeating [exams] at any stage” (TS14), explained that exam failure is common in medical school. The majority (8/15) of participants repeated a year (one even two years) due to exam failure. A few (2/15) found the rules to pass exams too punitive:

“Even if 80% of the class fails, they [lecturers] think that’s the best exam. You know it’s so discouraging. It’s frustrating. Sometimes you rise to your best, you answer everything as they were given to you, and you still fail.” (JD7)

A male doctor recalled being bullied by a male lecturer after the entire class failed an exam:

“There was this test we had and all of us failed in class. And the lecturer came and *he* was just saying we are not serious, we didn’t read, we didn’t listen, some of us don’t come to class. That day I was not motivated to read. I went home, watched movies, slept…the days in which I thought we had been bullied by any lecturer I didn’t read.” (JD2)

##### Culture of perceived unfairness

Rumours of impartiality in scholarship offers, and perceptions of favouritism in class and examination, hinted towards a culture of unfairness at the medical school.

*Rumours of impartiality in scholarships*

A couple of doctors (2/15) highlighted impartiality in the scholarship granting process:

“A lot of people [medical students] apply for them [Government grants]. And not everyone gets it…Even the grant giving office, there’s some corruption there. There’s some who get the grant before others by own means.” (JD1)

“If you know people in the Ministry, those people who give the scholarships, then you have a chance [of getting a scholarship].” (JD5)

*Perceptions of favouritism in examination*

Few junior trainees (2/15) heard rumours about ‘connections’ and bribery being reasons why some students might more easily pass exams than others:

“In medical school we used to hear ‘ok this person was going to this lecturer to give money’ or ‘this person’s parents called this lecturer’. We hear it a lot.” (JD9)

Another doctor (JD11), however, had never heard of bribery occurring at the School. Although none of the participants reported having experienced bribery first hand, according to two junior interviewees (2/15) another story that circulated the College, was that exam outcomes could be negatively influenced if students have a confrontation with a lecturer.

“Like maybe they [students] had, probably outside of the College, a confrontation with a lecturer and then come into the College and then they [students] find that it could be very difficult to get passed [for the exam]. And…we heard about sexual harassment. Maybe lecturers going after female students and then when you don’t comply it gets very difficult to pass as well.” (JD10)

Lecturers (2/7) agreed that clinical assessments were subjective at times. One (TS14) explained this was due to lack of standardisation of assessment questions.

Several junior doctors (3/15) raised the concern that there is no formal process where these perceptions of unfairness in the examination of undergraduate medical students can be taken to and reported and/or redressed: “The moment you report [favouritism in examination], you’ll be suppressed.” (JD13)

#### Theme 4. Coping strategies

Various coping strategies were identified during the interview study with reference to school, staff and student levels.

##### School level

At the medical school level two coping strategies could be distinguished.

*‘Creatively’ hire extra teaching staff*

According to teaching staff (6/7) and junior doctors (9/15) additional personnel were ‘creatively’ hired to increase the teaching workforce of the school. Four different approaches could be identified in the analysis. First concerns the hiring of international lecturers.

“In the premed [premedical education] yes it was quite normal [to have international lecturers] because we didn’t have any physiology teacher [in Sierra Leone]. The one who we had went away for studies so they had to step in. And we also had one [Country x] [lecturer], in third year. But I think those were the only two we had [when] foreigners [were] teaching.” (JD10)

Hiring foreign lecturers, however, was not regarded a sustainable solution, according to one faculty staff member (TS12) because it is expensive and they only come for “short periods”.

The second approach is the “Government bring back the diaspora appeal” (TS17), which was implemented post-conflict to encourage Sierra Leonean (health) professionals who migrated during the war to return.

A third way to increase the medical teaching staff is the rehiring of retired local clinicians on voluntary contractual basis.

“It is it’s quite common [to rehire retired doctors] because we are short of staff. I personally came back…So it was in fact the President [of Sierra Leone] who awards these contracts for reemployment.” (TS20)

Fourth and final strategy that could be identified is that the Institution sought teaching support from senior students and house officers: “You know but one thing I’m doing now is spotting talents in junior doctors and encourage them to be able to teach” (TS10).

Half of junior doctors (7/15) were involved in teaching medical students either in a formal role as Demonstrator or Associate Lecturer at the College (5/15) or more informally (2/15) by advising students during ward rounds. Once they successfully graduated they could be formally hired by the School. Two (2/15) already started teaching informally towards the end of medical school: “Because in final year I used to demonstrate clinical skills to the fourth year” (JD4).

Only one of the seven junior doctors involved in teaching did an education course. Others relied on being mentored by senior teaching staff and their provision of teaching materials:

“And of course I, in fact we [senior teaching staff] are setting up a communication and interpersonal relationship [with junior teaching staff]. He [the junior doctor] did it. I gave him all the materials.” (TS10)

Although one junior doctor (1/15) found that medical students “can relate better with you than with the consultant” (JD2), others (2/15) and a senior lecturer (1/7) articulated the view that using less experienced staff to teach students was not a good idea:

“Anatomy we had a few teachers but some were either too busy to teach or some were not really lecturers, maybe assistant lecturers or volunteers going to teach us anatomy. Which wasn’t a very good idea because…passing the message can be difficult if you’re not really trained.” (JD10)

*Base teaching schedule upon availability of teaching staff*

The second coping strategy employed by the medical school was to develop a teaching schedule that was subject to the availability of lecturers. Staff members at COMAHS (2/7) explained that the yearly curriculum, the types of subjects taught and the order in which they are taught, are guided by availability of teaching staff.

“The curriculum [at COMAHS] was in themes [in 1998]…But about two or three years afterwards we noticed that because of the paucity of lecturers we could not continue that. So we had to change the curriculum…to the classical subject-based curriculum.” (TS16)

Consequently, as highlighted by the same lecturer, medical topics were not always taught in a logical order. In addition, some subjects were not taught at all, which was the case for medical ethics for some years according to interviewed junior doctors.

##### Staff level

Another two coping strategies were identified at the medical school staff level.

*Juggle multiple roles*

Lecturers working for COMAHS tend to deal with the associated pressure and limitations by juggling multiple roles (teaching, clinical work in public and/or private sector, admin etc.), which was “hectic” (TS14) and “challenging” (TS12) as described by some (2/7) lecturers interviewed.

One way clinicians coped with juggling competing roles was having students attend their private clinics for teaching purposes: “Here [in my private clinic] I spend more time with them [the students] than in the lectures” (TS10).

*Teach flexibly*

Another coping strategy teaching staff seemed to apply was to teach medical students flexibly. This strategy was reported by few (2/15) junior doctors. One (JD11) stated that if lecturers were unable to teach during the week they might organise a Saturday class. Another reported staff ‘bundling’ lectures:

“Some lecturers don’t always come on time. Maybe they don’t show up for quite some time. And the time they come they would just bundle everything maybe in a few lectures which should have been done over a few weeks.” (JD14)

##### Student level

At student level six coping strategies were highlighted during the interviews.

*Comply with unwritten rules*

From accounts by a couple of junior doctors (2/15) it seemed that hard work and conflict avoidance appeared to be the unwritten rules to survive medical school, in other words ‘playing the game’:

“So I did hard work, relentlessly. Obey all rules and regulations to the best of my ability. Avoid any conflict or confrontation with any lecturer. Try to be in the good books. You know those attitudes you need to definitely have in a way.” (JD7)

*Negotiate teaching support from less qualified health personnel*

Another way how junior doctors (7/15) got by as students was to ask less qualified health staff to teach, which was particularly necessary for bedside teaching:

“The consultant actually usually comes for lectures. For bedside teaching it was very rare. We could have maybe a consultant once a week, twice, once in two weeks. Mostly they were house officers, medical officers just around. They were doing the bedside teachings with us.” (JD10)

Junior doctors (7/15) remembered being taught by a variety of staff, e.g. house officers, demonstrators, medical officers, nurses, midwives, volunteers and senior medical students:

“Even when we came to the clinical years, which is [includes] more teaching, we’ve been taught by immediate senior colleagues [medical students one or two years ahead of us].” (JD9)

*Get by with self-directed learning*

The majority (8/15) described the reality of this learning style suggested by the medical school:

“You have to do extra work. Extra, extra, extra work, because they [the lecturers] can’t complete the syllabus. So it’s you who has to do that extra reading.” (JD15)

One of these eight junior doctors even recalled some subjects being largely self-taught:

“Because I could remember when we were in the first year. [Subject X] was done mainly by ourselves. We read from the textbooks and then come and apply it. [Subjects X and Y]: we learned them by ourselves.” (JD9)

*Cooperate with other students*

Four junior doctors (4/15) highlighted working together with fellow students to improve their learning: “You have to do the studies. So in our class we almost organise ourselves in some groups. Study in the afternoon” (JD14). One added that students helped each other to gain access to textbooks:

“Economically and socially because you don’t have the time to go out to fend for yourself…Sometimes that’s more the reason why it was important for us to be in groups. We benefitted from those colleagues whose parents could afford it [Textbooks]…So we used to link up with those colleagues and photocopy you know some of those things. And that’s how we had to survive.” (JD8)

*Student union acting as an* advocate for *students*

A minority (3/15) spoke about the Student Union being an advocate for students when they experienced difficulties at medical school.

“So sometimes students pay fees for certain facilities. And those facilities are not being offered to students. For example, for many years we paid for ICT, for technology, computer services. But there were no computer services available. So [the Student Union] had to talk to the administration and say we don’t pay or we pay and those facilities are being made available” (JD5)

*Rely on financial and moral support from family and friends*

Many (12/15) relied on the financial support from family and/or friends within Sierra Leone and the diaspora to pay for the medical study, including tuition fees (for the years they did not receive a scholarship), ‘additional charges’ by the College (like laboratory, sports, computer services), textbooks, photocopying, food, transportation and other important necessities.

“We are managing [financially], friends, family, yeah. But it wasn’t easy because my mother is alone. She has to take care four of us so it wasn’t easy for her. But thank God through the help of friends’ parents, and then my family.” (JD15)

“Well especially the materials, the textbooks and all the rest of it, the majority of them are not here for sale. Except I mean if you have someone who has family in England and all the rest of it. Your uncle or your aunty and they say buy these medical books for me.” (JD11)

A few (3/15) participants talked about ‘jobs on the side’ to cope financially:

“From the time I lost my dad, I took over the full responsibility for myself. So in medical school sometimes intermittently I leave school but abandon it. Like a week when I know this time they [the medical school] are not going to deliver so many lectures…Then I would do some part-time job.” (JD6)

Family and friends also served as a source of moral support, which was reported by several (3/15):

“It was the dream of my mum for me to become a doctor. So she’s been with like me throughout all of it. And she used to sit with me during exams when I was studying at night. And she’d be sleeping by the table, just to make sure that I was studying.” (JD4)

## Discussion

This examination of undergraduate medical education experiences in Sierra Leone has revealed several interesting areas for discussion. The capacity issues identified by interviewees are typical for medical schools in low income settings, and therefore are not surprising. Physical, human, and financial capacity challenges are particularly common in medical education institutions in the SSA region [14, 15, 36, 37] which was reflected in our results.

Interviewees reported a need for more lecturers. Ideally these should be working full-time and Sierra Leonean, whom are generally more knowledgeable about the local needs and context and more likely to stay in the country long-term than tutors from outside the country. Besides quantity, results suggest that the quality of the teaching workforce at the MBChB programme requires strengthening, as also highlighted in the current Human Resources for Health Strategy [10]. Moreover, lecturers at COMAHS need to be offered fair reimbursements for their work, which accounts by interviewed staff have shown is not always the case. Professional development and salaries are recognised motivators for health workers [38]; therefore, making improvements in these areas for educational/clinical staff at COMAHS may positively affect their motivation about being part of the college.

While our findings suggest that more trained Sierra Leonean medical educators are required, this will likely not be achieved in the short run. Interviewees note that staff and students are finding resourceful ways to deal with faculty shortages. For example, junior doctors with an interest in and an affinity for teaching are encouraged to bridge the gap in lecturers. Additionally, students seem to rely heavily on peer-led and self-directed learning. Participants suggested that having a clear and readily accessible syllabus with adequate learning resources would further strengthen these adaptive learning strategies.

Poor physical capacity was a common complaint, especially with regards to infrastructure and learning resources. The library at the campus in central Freetown had a range of books but junior doctors commented these were mostly outdated. Students had minimal access to relevant teaching aids and clinical skills equipment, and few had access to ICT facilities or the Internet. Mullan et al. (2011) found similar challenges in their survey of senior medical faculty across SSA [37], as did Ethiopian medical students when their medical school intake rapidly increased (to address national physician shortages) without adequate investment in physical infrastructure or teaching resources [22]. But the difficulties reported by interviewees in our study were particularly stark. Even basic utilities like electricity, water, and food were felt to be lacking, particularly at the Kossoh Town campus on the outskirts of Freetown where first and second year students were based. The importance of basic needs like food, water and comfort to learning are well recognised [39], and it is easy to see how learning can suffer if these needs are not met.

Since this study was conducted (i.e. end of 2013) the medical school in Sierra Leone has addressed a number of important issues. There has been significant investment aimed to improve the infrastructure and staffing levels. Funding was obtained to bring in senior academic and clinical staff from outside of the country to strengthen the College and the teaching hospitals where students participate in clinical placements. Some clinicians have participated in specialist training in education and further staff development is being planned. A library is now functioning at the University campus where the first years of the curriculum are delivered and a clinical skills training room has been opened at Connaught Hospital (the main clinical site).

Whilst the above-mentioned issues relating to resource limitations are common in the SSA literature on medical education, the theme related to stress is less well researched. Interviewees in our study reported an enormous pressure to succeed in their medical education. This pressure, together with minimal pastoral and academic support, and a lack of alignment between learning objectives and assessment methods produces a perfect storm for potential student burnout. High stress amongst medical students is well recognised in the literature from higher-income settings [40, 41], and can contribute towards dropout and academic failure [42]. The attrition rate over the 6-year medical course at COMAHS was estimated at 45% [12] and represents a desperate waste of talent and investment in a country with one of the lowest doctor-population densities in the world. To put this into context, an international literature review found the average medical student attrition rate was 11% (range 2.4–26.2%), with the only country included from SSA (South Africa) reporting a 16.7% dropout rate [13]. Whether the stress levels at COMAHS cause the high attrition rates or vice versa is not yet clear and requires further investigation.

Recent studies from Nigeria linked higher stress levels amongst undergraduate medical students to inadequate educational resources, family pressure and financial difficulties but did not discuss attrition rates [43, 44]. Medical graduates interviewed in a South African study also noted the impact that exams and patient exposure had on them emotionally, and the need for support mechanisms, but did not comment on attrition rates either [22]. Interestingly the 2011 SSA Medical Schools Study did not identify student stress or dropout to be significant challenges in the region at all [15, 37]. This could be because the perspectives of students and graduates were not included in their study. However the Ethiopian [22] and Kenyan [23] studies looking at student experience did not identify stress or burnout as important issues either. Whether this is due to differences in: study methodology; openness of interviewees; resource limitations; course design; or academic rigor, is unclear. What is clear from this study is that stress was a shared experience of medical school in Sierra Leone amongst participants and that it should be taken seriously.

The examination process was viewed by junior doctors as particularly stress inducing due to its perceived difficulty, irregularity and unfairness. Green-Thompson (2012) also found the lack of feedback and perceived unstandardised nature of assessment at a South African medical school exacerbated stress and anxiety [21]. Some of these issues are already being addressed by COMAHS in the revised 2014 MBChB curriculum [18]. For example, this new curriculum describes more regular exams in the Basic Sciences; at the end of each semester instead of the school year. A semester-based system may reduce stress, as students are examined about less material at a time.

Teaching by humiliation and mistreatment is widespread in medical schools and was a common theme throughout our interviews. Previous research on undergraduate medical education has revealed similar findings in low and middle-income settings [45–48] and high-income countries [49–51]. Such teaching methods may have contributed towards the high stress levels reported. This is supported by a recent study amongst Sierra Leonean university students that showed issues with instructors to be a cause of stress [52]. The literature on mistreatment of medical students and junior doctors highlights how difficult it is to change what is often a cultural or institutional norm [53]. Successful change requires long-term, sustainable efforts to reduce the power imbalance between students and educational/clinical staff and support students to be more assertive [50].

### Limitations

This study has several limitations which should be taken into account. Firstly, this study only includes the views and experiences of junior doctors who successfully completed their undergraduate medical education in Sierra Leone. This means the views of those who dropped out are not included. Future research should consider to explore the perspectives of such students. Secondly, results are based on the views of a limited and purposively selected sample of junior doctors and teaching staff; consequently, there is some degree of selection bias and results cannot be generalised to the entire medical student or teaching staff population at COMAHS. Still this exploratory and in-depth study provides a solid basis for further research. An area of concern that particularly requires examination is the prevalence and severity of stress amongst medical students in Sierra Leone and its relationship to medical school drop-out. Thirdly, there may have been recall bias as most junior doctors had graduated for several years from medical school before being interviewed for this study. Future research could overcome this obstacle by focusing on medical students. Lastly, the coding framework was developed and applied by a single researcher. Although the framework was discussed with project supervisors to minimise reliance on the interpretation of results by one person, this may have introduced bias.

## Conclusions

This study is the first to explore in depth the experiences of recent graduates from Sierra Leone’s only medical school. In keeping with the existing literature on medical education in SSA, concerns regarding the adequacy of educational resources, staffing and infrastructure were commonly raised. Our study however offers new insights into less well reported challenges in this region. The lack of formal curricula or feedback on performance made it difficult for students to know how to prepare for their rigorous end of year exams. This under-preparedness combined with a pressure to succeed, difficult living conditions, and limited access to educational resources or guidance, created a stressful learning environment. These issues are under-recognised in the existing SSA medical education literature. Understanding and improving the student experience is an essential step towards increasing the number of doctors in Sierra Leone. COMAHS has already begun to address some of the challenges by introducing new curricula and assessment methods with assistance from international partners.

## Additional file

Additional file 1: Topic Guides. This document contains the topic guides used for interviews with junior doctors and teaching staff. (DOCX 23 kb)

## Abbreviations

COMAHS: College of Medicine and Allied Health Sciences
JD: junior doctor
MBChB: Bachelor of Medicine and Bachelor of Surgery
MoHS: Ministry of Health and Sanitation
SSA: Sub-Saharan Africa
TS: senior teaching staff
WASSCE: West Africa Senior Secondary School Certificate Examination
