# Supplementary material for: Undergraduate medical education in Sierra Leone: a qualitative study of the student experience
Source: BMC Med Educ. 2018 Dec 7;18:298. doi: 10.1186/s12909-018-1397-6 (PMC6286603; doi:10.1186/s12909-018-1397-6)
Supplement: Supplementary file 1 — Topic Guides. This document contains the topic guides used for interviews with junior doctors and teaching staff. (DOCX 23 kb) [file 12909_2018_1397_MOESM1_ESM.docx]

**Topic guides**

**Junior doctors**

Before the interview

- Thank you for agreeing to participate in this study on medical training in Sierra Leone.
- Was everything clear in the information sheet and consent form? Understand that you can stop participating at any time? Understand how quotes arising from this interview will be used?
- Any questions before we start?
- If you don’t mind I will start the audio recorder now so that I can concentrate fully on what you are telling me. *[If they have agreed to allow recording of the interview then start recording now, otherwise take notes]*

Life-line

*[At the start of the interview I’ll ask participants to fill in the life-line. Briefly explain. Afterwards there is opportunity to briefly discuss what they’ve written.]*

Topics and sample questions (relevant to data used for this paper)

**Experiences of undergraduate medical education**

1. When did you start your education at COMAHS? And when did you graduate?
2. Why did you choose to do your medical education at COMAHS? *Probe: Are there any other (private) medical training institutions in Sierra Leone? Are there opportunities abroad you considered?*
3. What were your experiences as a medical student? *Probe: what did you enjoy most? What did you enjoy least? Experience with teaching staff/course materials/teaching environment*
4. What were the main challenges you faced during that time? Why?

**Financing** **of medical education**

1. Is medical education expensive in Sierra Leone?
2. If I may ask, how did you fund your medical education? *Probe: self-funding/scholarship/government/family*
3. How did you cope financially as a medical student? *Probe: any challenges (e.g. fees, housing, living)?*

End of interview

- Thank you for your time and effort and contribution to this study
- *[If still need more participants]* Can participant recommend any other stakeholders? If so what is his/her name and email address (for confidentiality purposes I’ll not disclose it was you recommending him/her)

**Teaching staff COMAHS**

Before the interview

- Thank you for agreeing to participate in this study on medical training in Sierra Leone.
- Was everything clear in the information sheet and consent form? Understand that you can stop participating at any time? Understand how quotes arising from this interview will be used?
- Any questions before we start?
- If you don’t mind I will start the audio recorder now so that I can concentrate fully on what you are telling me. *[If they have agreed to allow recording of the interview then start recording now, otherwise take notes]*

Topics and sample questions (relevant to data used for this paper)

**Role/background**

1. Could you start off by telling me a bit about your work at COMAHS? *Probe: since when working there, role, changed over time*
2. In your opinion, what are the main challenges at COMAHS?

**Quality of medical education**

1. How would you describe the quality of undergraduate medical education provided at COMAHS?
2. In what areas do you think quality of education could be improved? And how can this be achieved in your opinion?

**Financing of medical education for COMAHS and students**

1. Do you get paid for your work at COMAHS? *Probe: sufficient or not*
2. Do you know how medical students fund their medical education? *Probe: self-funding/scholarship/government, family involvement, conditions (pay back)*

End of interview

- Thank you for your time and effort and contribution to this study
- *[If still need more participants]* Can participant recommend any other stakeholders? If so what is his/her name and email address (for confidentiality purposes I’ll not disclose it was you recommending him/her)
